# Grundriss der pathologischen Anatomie

**Published:** 1896-12

**Authors:** 


**Grundriss der pathologischen Anatomie.** Von Prof. Dr. R. Langerhans. Zweite vermehrte und verbesserte Auflage. Pp. x., 566. Berlin: S. Karger. London: Williams and Norgate. 1896.

This is an enlarged and improved version of the first edition of the book. The author modestly disclaims originality for his work, which is not designed to replace the larger and more comprehensive treatises on Pathology, but to present to the student and learner in pathology the main facts of the science in as comprehensive a manner as is consistent with a treatment of the subject within a moderate compass. There is no doubt that the book is well written, and proceeds from one who has a thorough mastery of his subject and is therefore well qualified to teach it. It is essentially a pathological anatomy; although the physiological side is entered upon when necessary, this is a subsidiary feature of the work. On most of the subjects treated, a good description, short but containing the essential points, of the pathological changes is found. The author has throughout avoided controversial subjects and the statement of conflicting views. The enumeration of pathological conditions with only a brief explanation—a necessary procedure to keep a book on pathology within moderate limits—is apt to render some parts of the subject difficult, especially to beginners.

The arrangement of the book is novel, and not altogether to be recommended. The author has departed from the plan usually followed in works on pathology of a division into a general and a special part, and thus saves some repetition at the cost of convenience of description. For example, under the head of Tuberculosis we find all the lesions associated with the presence of the bacillus in the various organs; and in the same chapter, on "diseases brought about through vegetable parasites, including the infectious diseases," the description of the changes in the lungs in pneumonia. Though this may be scientifically correct, it seems better to adopt the usual plan of treating the pulmonary lesions in tuberculosis and in pneumonia under diseases of the lungs and so on. As another example, the account of dysentery is given under diphtheria. Then the group of infective granulomata, well characterised by their anatomical changes, finds no place apart, these diseases being considered in the chapter above mentioned. The place of sarcomas in the chapter on histioid tumours also appears inappropriate. The chapter on diseases of the nervous system is hardly adequate to the subject, and suffers from the want of a few good diagrams. Inflammation and fever are also shortly dealt with. On the other hand, most sections of the book are very good.

The illustrations throughout are of moderate excellence. Taken as a whole the work presents a sound and scientific account of pathological anatomy, and the author has succeeded in giving a very large amount of information in a volume of moderate dimensions.

